# The development of the Screening of Visual Complaints questionnaire for patients with neurodegenerative disorders: Evaluation of psychometric features in a community sample

**DOI:** 10.1371/journal.pone.0232232

**Published:** 2020-04-29

**Authors:** Famke Huizinga, Joost Heutink, Gera A. de Haan, Iris van der Lijn, Fleur E. van der Feen, Anne C. L. Vrijling, Bart J. M. Melis-Dankers, Stefanie M. de Vries, Oliver Tucha, Janneke Koerts

**Affiliations:** 1 Department of Clinical and Developmental Neuropsychology, University of Groningen, Groningen, The Netherlands; 2 Royal Dutch Visio, Centre of Expertise for blind and partially sighted people, Huizen, The Netherlands; Universidad de Leon, SPAIN

## Abstract

**Background and objectives:**

Patients with neurodegenerative disorders often experience impairments in visual function. In research and clinical care, visual problems are primarily understood as objective visual impairments. Subjective complaints, referring to complaints from a patient’s perspective, receive less attention, while they are of utmost clinical importance to guide assessment and rehabilitation. A 21-item Screening of Visual Complaints questionnaire (SVC) was developed for the assessment of subjective visual complaints in patients with neurodegenerative disorders. This prospective study aims to evaluate the psychometric properties of the SVC in a large community sample.

**Methods:**

A stratified convenience sample of 1,461 healthy Dutch participants (18–95 years) without severe self-reported neurological, ophthalmological or psychiatric conditions completed the SVC, Cerebral Visual Complaints questionnaire (CVC-q), National Eye Institute Visual Function Questionnaire–25 (NEI-VFQ-25), Behavior Rating Inventory of Executive Function-A (BRIEF-A), Questionnaire for Experiences of Attention Deficits (*Fragebogen erlebter Defizite der Aufmerkzamkeit*; FEDA), Depression Anxiety Stress Scale–21 (DASS-21) and the Structured Inventory for Malingered Symptomatology (SIMS) online. After two weeks, 66 participants completed the SVC again. We evaluated the factor structure, internal consistency, convergent and divergent validity, and test-retest reliability of the SVC.

**Results:**

The sample was split in two subsamples to perform exploratory and confirmatory factor analyses. In the first subsample, the exploratory factor analysis extracted three factors from the SVC: *diminished visual perception*, *altered visual perception* and *ocular discomfort*. The confirmatory factor analysis showed this model to be valid in the second subsample. The SVC showed satisfactory convergent validity (NEI-VFQ-25: r = -0.71; CVC-q: r = 0.84) and divergent validity (SIMS: r = 0.26; BRIEF-A: r = 0.29; FEDA: r = 0.40; DASS-21: r = 0.34) and good internal consistency (Cronbach’s alpha = 0.85) and test-retest reliability (ICC = 0.82).

**Conclusions:**

The SVC is a valid and reliable tool for the assessment of subjective visual complaints in a community sample and appears promising for clinical use in patients with neurodegenerative disorders.

## Introduction

It is increasingly recognized that neurodegenerative disorders, including Parkinson’s disease (PD), multiple sclerosis (MS) and dementia, are characterized by impairments in visual function. Approximately 75% of PD, 33% of MS and 31% of dementia patients suffer from visual impairments [1–4]. When using objective visual function tests, a range of impairments can be identified in these patients, for example in visual acuity, contrast sensitivity, color discrimination, object localization, depth perception, motion perception or perceptual organization [2,4–8]. The visual system is of utmost importance in performing daily-life activities, such as reading, walking or performing household chores. Patients suffering from visual problems may therefore be hampered in daily life, which in turn can affect their quality of life [4,9–11].

In research and clinical care, visual problems are primarily understood as objective visual impairments. Objective visual impairments are impairments that are objectified by visual function tests of, for example, visual acuity or the visual field. Subjective visual complaints, on the other hand, often receive less attention than objective visual impairments. Subjective visual complaints are patient-oriented descriptions of visual disturbances experienced in daily life, either at a functional level with a description of the vision itself (e.g. unclear vision or double vision) or at an activity level (e.g. difficulties with reading or watching television). Subjective complaints are usually less clearly defined than impairments identified with objective measures. PD or MS patients, for example, can report their vision as being ‘blurred’, ‘washed out’ or ‘distorted’ [12,13]. As useful as objective measures are for the assessment of visual impairments, it remains unclear whether they fully reflect the degree of visual problems patients experience in daily life [14–17]. An accurate and complete view on the difficulties patients encounter in daily life is of utmost clinical importance in order to guide further assessment, care and rehabilitation [14]. Therefore, clinical instruments aiming at the assessment of patients’ complaints and related difficulties in daily life are of critical relevance.

Despite the relevance of such instruments, there is a lack of measures that can be used to assess subjective visual complaints. Patients with neurodegenerative disorders who suffer from visual problems might therefore not receive the care and rehabilitation they need. The instruments that are currently available are primarily aimed at assessing vision-specific health-related quality of life in daily activities and do not fully capture subjective visual complaints at a functional level [14]. One of these questionnaires is the 25-item National Eye Institute Visual Function Questionnaire (NEI-VFQ-25). The NEI-VFQ-25 is a widely used questionnaire for the assessment of vision-related quality of life in patients with chronic eye diseases [18]. It aims to assess the impact of visual symptoms on daily-life activities, such as reading street names or watching a movie, and multiple dimensions of health-related quality of life, including emotional well-being and social functioning [18]. The NEI-VFQ-25 was found to be valid for the assessment of vision-related quality of life in different patient samples, including MS [19–22] and PD [23,24], but is unfortunately limited in that it does not assess subjective visual complaints at a functional level.

A questionnaire that is aimed to measure subjective visual complaints at a functional level is the Cerebral Visual Complaints questionnaire (CVC-q). The CVC-q originates from the Cerebral Vision Screening questionnaire developed by Kerkhoff, Schaub and Zihl [25]. This originally 10-item questionnaire aims to measure visual complaints in patients with acquired brain injury and was found to be valid in individuals following stroke [26]. For application in visual rehabilitation centers, this questionnaire has been modified to the 43-item CVC-q. The CVC-q captures visual complaints at a functional level, such as unclear vision or an altered experience of color, as well as problems in vision-related activities, such as reading or climbing stairs, This amplification of the questionnaire serves a more thorough evaluation of problems patients encounter in daily life in order to guide specialized visual care and rehabilitation. Although clinicians find the CVC-q useful to guide rehabilitation in visual rehabilitation centers, a practical disadvantage is that the CVC-q contains a large number of items and is developed to be applied by a trained healthcare professional. Administration of the CVC-q is therefore time-consuming, making it unsuitable for use by medical specialists including general practitioners and neurologists, who often have relatively short consultation meetings per patient. Consequently, there is a great need for a short self-report patient-centered screening tool that (1) can be used by medical specialists, (2) helps recognizing subjective visual complaints often reported by patients with neurodegenerative disorders, and (3) ultimately improves referral of visually impaired patients to specialized care and treatment.

For this purpose, the Screening of Visual Complaints (SVC) questionnaire was developed. The SVC has been partly derived from the CVC-q and was developed by clinical experts working with patients with neurodegenerative disorders including PD and MS. Central in the development of the SVC were the experiences from PD and MS patients themselves, as well as input from an expert group and the literature. The questionnaire consists of 21 items, with 19 items reflecting subjective visual complaints at a functional as well as at an activity level. It includes visual complaints that are often reported by patients with PD or MS, such as an altered perception of objects or faces, seeing things that others do not see, double vision, being blinded by bright light, seeing shaky, jerky or shifting images and missing part(s) of the visual field, which are not all being captured by the CVC-q or NEI-VFQ-25. In contrast to the CVC-q and the NEI-VFQ-25, the SVC is short in length and can easily be self-administered, which makes it a suitable instrument for use in clinical neurological and general practice. Psychometric properties of the SVC are currently unknown. Therefore, the present study aims to evaluate the psychometric properties of the SVC in a large community sample by examining its factor structure, internal consistency, convergent and divergent validity, and test-retest reliability. Furthermore, we aim to evaluate the relation between visual complaints and participation in daily life. We expect the SVC to show a good internal consistency, convergent and divergent validity, and test-retest reliability. In addition, we expect a moderate relation between the SVC and participation in daily life.

## Materials and methods

### Study design

This study had a cross-sectional design. See S4 Appendix for the checklist of the STARD guidelines (Standards for Reporting of Diagnostic Accuracy Studies) [27]. Since we did not assess the diagnostic accuracy of the SVC but other psychometric properties (i.e. convergent and divergent validity, factor structure, internal consistency and test-retest reliability), not all the STARD criteria were applicable or available to our research. We reported ‘not applicable’ or ‘not available’ for these criteria.

### Participants

Participants of 18 years and older were included in the study. Participants formed a stratified convenience sample, and were included systematically within age blocks of ten years, aiming for an equal distribution of participants among age groups. In addition, we aimed for an equal representation of educational levels and sex within age groups. Participants were excluded if they reported a severe neurological condition (e.g. MS, PD, traumatic brain injury, brain tumor, cerebrovascular accident, epilepsy, narcolepsy), ophthalmological condition (e.g. damage of the eye(s) or of the optic nerve), or psychiatric condition (e.g. schizophrenia or the presence of psychoses). The presence of these medical conditions was screened by means of self-report. Participants were asked: “Did you ever visit a neurologist?” (answer options yes or no), and, if yes: “For which neurological condition did you visit a neurologist?”. The same approach was applied to screen for ophthalmological and psychiatric conditions.

### Materials

#### Screening of Visual Complaints (SVC)

The SVC is a Dutch self-report questionnaire composed of 21 items (see S1 Appendix for the original Dutch questionnaire that was used in the present study; see S2 Appendix for an English translation). The SVC was developed by an expert group consisting of neurologists, neuropsychologists, ophthalmologists, occupational therapists and clinical physicists. All members of the expert group have ample experience in clinical care of patients with neurodegenerative disorders, with some being specialized in diagnosis and rehabilitation of visual impairments in these patients. For the development of a patient-centered questionnaire, involving the patients’ perspective is crucial since they are experts on their own subjective experiences [28]. Therefore, the development of the SVC was also based on experiences of PD and MS patients themselves. In this development, an old version of the CVC-q (pre CVC-q) functioned as the main starting point. To gather patients’ experiences, we analyzed responses on the pre CVC-q questionnaire that was administered in 28 PD and 33 MS patients visiting visual rehabilitation centers in the Netherlands in 2015 and 2016. The pre CVC-q contained one open-ended question asking respondents whether they experience visual complaints in daily life, and if yes, which visual complaints they experience. This open-ended question is followed by 8 structured items on visual complaints. The visual complaints the PD and MS patients reported on these items (i.e. the open-ended question and the 8 structured items) of the pre CVC-q questionnaire were explored to evaluate which complaints were frequently present and thus should be considered to be included in the SVC questionnaire. On the open-ended question of the pre CVC-q, PD patients often reported complaints of double vision, seeing things that others do not see, having problems with depth perception or estimating distances, and having trouble with reading. On the structured items of the pre CVC-q, frequently reported complaints in PD were unclear vision, having trouble seeing at reduced contrast, being blinded by bright light and needing more light to see things. In MS the most frequently reported complaints on the open-ended question of the pre CVC-q were double vision, seeing shaky, jerky or shifting images, having trouble reading, and missing part(s) of the visual field; on the structured items of the pre CVC-q, frequently reported complaints were unclear vision, being blinded by bright light, having difficulty adjusting to light or dark, and having trouble seeing at reduced contrast. Based on this exploration of patient reports, together with the input from an expert group and the literature, 7 items were included in the SVC that originated from the pre CVC-q questionnaire (SVC items 2, 8, 10–12, 19–20) and 12 new items were created for the SVC (SVC items 3–7, 9, 13–18).

Participants were instructed to answer the questions of the SVC based on their experience over the past weeks. If they wear glasses or contact lenses, they were instructed to answer the questions as though they were wearing them. The SVC starts with a semi-structured question asking respondents to indicate whether they experienced visual problems in daily life on a 3-point Likert scale ‘no/hardly ever’ (0), ‘sometimes’ (1) or ‘often/always’ (2) and, if present, to report the type of visual complaints they encounter. Thereafter, the 19 structured items follow. Participants can answer whether each of these complaints are present on a scale ‘no/hardly ever’ (0), ‘sometimes’ (1) or ‘often/always’ (2). The questionnaire ends with an item asking participants to rate their limitations in daily life due to the visual complaints mentioned before on a scale from 0 (no limitations) to 10 (very severe limitations). Furthermore, to facilitate the use of the SVC in clinical practice, the questionnaire includes questions asking for general information (date, name), demographic characteristics (sex, date of birth, educational level) and clinical information (visiting an ophthalmologist/ the presence of an ophthalmologic condition and whether or not participants would appreciate advice, assessment and/or rehabilitation for the visual complaints). The total score of the SVC is calculated by summing the scores of the 19 structured items of visual complaints and ranges from 0 to 38. Higher scores on the SVC indicate a higher frequency or severity of visual complaints.

#### Cerebral Visual Complaints questionnaire (CVC-q)

The CVC-q originates from the Cerebral Vision Screening questionnaire, developed by Kerkhoff, Schaub and Zihl [25] and modified by Dittrich [29]. The latter was translated into Dutch [29]. During the time the SVC was developed, the older version of the CVC-q, the pre CVC-q, has been modified to the 43-item CVC-q and 3 items that were newly developed for the SVC questionnaire were also added to the CVC-q (SVC items 4, 5, 6). Therefore, and since the SVC was partly based on the pre CVC-q, The SVC shares in total 10 out of 19 items with the CVC-q (7 items originated from the pre CVC-q questionnaire, and 3 newly developed items were added to the SVC and the CVC-q questionnaires). The CVC-q focuses on the presence and severity of subjective visual complaints or visual difficulties in daily-life activities in patients with acquired brain injury, such as reading or mobility. Although the validity and reliability of the CVC-q are not yet known, the Cerebral Vision Screening questionnaire, where the CVC-q is based on, was found to show good convergent validity (correlation coefficient of 0.64) and good interrater test-retest reliability (correlation coefficient of 0.76) in individuals following stroke [26]. The internal consistency of the Cerebral Vision Screening questionnaire was not evaluated [26].

The CVC-q consists of 16 overarching items that are answered by all participants and are measured on a 3-point Likert scale with the answer options ‘no/hardly ever’ (0), ‘sometimes’ (1) or ‘often/always’ (2), with the exception of two items which are answered with ‘yes’ (2) or ‘no’ (0). In the present study, a total score on the CVC-q was calculated by summing the scores of the 16 overarching items. The remaining 27 items are supplementary, of which 24 items are only completed when participants answer ‘sometimes’ or ‘often/always’ to the overarching item to which the supplementary item is related. Of the other three supplementary items, two are open-ended questions and one question specifies an overarching item. The supplementary items further specify and qualify visual problems in order to guide visual rehabilitation, and were for that reason not included in the calculation of the total score. The total score of the CVC-q ranges from 0 to 32, where a higher score indicates a higher frequency or severity of visual complaints.

#### National Eye Institute Visual Function Questionnaire– 25 (NEI-VFQ-25)

The NEI-VFQ-25 is a 25-item self-report questionnaire and is a short version of the 51-Item National Eye Institute Visual Function Questionnaire [30,31]. The questionnaire measures the impact of visual symptoms on daily functioning, emotional well-being and social functioning [18]. The NEI-VFQ-25 has a good convergent validity (correlation coefficients of the subscales exceeding 0.90 with the original 51-item version) and a good internal consistency (Cronbach’s alphas of the subscales ranging from 0.71 to 0.85) in patients with various chronic eye conditions [18]. The test-retest reliability was not assessed [18]. The NEI-VFQ-25 consists, next to the original 25 items, of an additional 13 items. These additional items were added as an appendix based on recommendations of the authors to have a more accurate indication of patients’ visual function. All items together represent eleven vision-related constructs and a general health construct. The items were scored on a 5-point Likert scale with the scores 0, 25, 50, 75 or 100, with the exception of one item which was scored on a 6-point Likert scale with the scores 0, 20, 40, 60, 80 or 100, and two items from the appendix which were scored on a scale from 0 to 10 and subsequently converted to scores from 0 to 100. Item scores resemble the achieved percentage of the total possible score in visual quality of life. Total scores on the subscales general health, general vision, near activities, distance activities, social functioning, role difficulties, dependency, mental health, driving, peripheral vision, color vision and ocular pain were calculated by averaging the scores on the relevant items. In addition, the total score on the NEI-VFQ-25 was calculated by averaging the scores of all subscales, given equal weight to each subscale. A higher score indicates better visual quality of life.

#### Behavior Rating Inventory of Executive Function—Adult (BRIEF-A)

The BRIEF-A is a self-report questionnaire that measures executive functioning or self-regulation in an everyday environment [32,33]. The questionnaire has a good internal consistency (Cronbach’s alpha of 0.96) and test-retest reliability (intraclass correlation coefficient of 0.77) in a community sample of Dutch participants, and a good convergent validity in a clinical sample of patients with autism (correlation coefficient of 0.63) [32]. The BRIEF-A is composed of 75 items that measure two constructs: metacognition and behavioral regulation. Items were measured on a 3-point Likert scale with the answer options ‘never’ (1), ‘sometimes’ (2) or ‘often’ (3). The total score of the BRIEF-A and the scores on the two constructs were calculated by summing the scores for the relevant items. A higher score indicates poorer executive functions in everyday life. Furthermore, the BRIEF-A contains three scales which can be used to check the validity of answers: negative tendency, improbability and inconsistency. To ensure validity of answers, participants were excluded when they scored above the cut-off as defined in the manual on any of these three scales (negative tendency>3, improbability>2, inconsistency>7) [32].

#### Questionnaire for experiences of attention deficit

The Questionnaire for Experiences of Attention Deficit (*Fragebogen erlebter Defizite der Aufmerkzamkeit*; FEDA) is a self-report questionnaire consisting of 27 items measuring attentional deficits [34]. The FEDA has a good internal consistency (Cronbach’s alphas of subscales ranging from 0.88 to 0.94) and a moderate convergent validity (correlation coefficients of subscales ranging from 0.18 to 0.40 with objective tests) in patients with acquired brain injury and healthy controls [34]. The test-retest reliability was not assessed [34]. Items were scored on a 5-point Likert scale with the answer options ‘never’ (1), ‘rarely’ (2), ‘sometimes’ (3), ‘often’ (4) or ‘very often’ (5). The total score was calculated by summing the scores for the items. Higher scores indicate poorer attentional functions.

#### Depression Anxiety Stress Scale– 21 (DASS-21)

The DASS-21 is a 21-item self-report instrument designed to measure the constructs of depression, anxiety and stress [35]. The questionnaire has a good internal consistency (Cronbach’s alphas of the subscales ranging from 0.89 to 0.96) and a good test-retest reliability (correlation coefficients of the subscales ranging from 0.71 to 0.81) in a clinical sample of patients with anxiety [36]. Convergent validity was not assessed in this original study, but was found to be good (correlation coefficients of the subscales ranging from 0.47 to 0.66) in a recent study in healthy participants [37]. Items of the DASS-21 were scored on a 4-point Likert scale with the answer options ‘never’ (0), ‘sometimes’ (1), ‘often’ (2) or ‘very often’ (3). Each construct contains seven items. The total score of the DASS-21 and the construct scores were calculated by summing the scores for the relevant items. A higher score indicates more severe symptoms.

#### Structured Inventory for Malingered Symptomatology (SIMS)

The SIMS is a self-report instrument that screens for simulation or exaggeration of psychiatric symptoms [38,39]. The questionnaire has a good internal consistency (Cronbach’s alpha of 0.72), a good test-retest reliability (correlation coefficient of 0.72), and a moderate convergent validity (correlation coefficient of 0.33) in college students [40]. The SIMS consists of 75 items with the answer options ‘yes’ or ‘no’. Depending on the positive or negative formulation of an item, either ‘yes’ or ‘no’ was scored with 1. The total score was calculated by summing the scores of the items. A higher score indicates more simulation or exaggeration of psychiatric symptoms. In addition, the total score was used to evaluate the validity of answers. Participants who obtained a total score of 17 or higher, indicative of overrepresentation or simulation of symptoms, were excluded.

#### Utrecht Scale for Evaluation of Rehabilitation-Participation (USER)

The USER is a self-report questionnaire that aims to measure level of participation in daily life [41]. It comprises 21 items measuring three constructs of participation in daily life: frequency, restrictions, and satisfaction. The USER has a good internal consistency (Cronbach’s alphas of the subscales ranging from 0.70 to 0.91) and convergent validity (correlation coefficients of the subscales ranging from 0.59 to 0.75) in persons with physical disabilities [41]. The test-retest reliability of the USER was not assessed in this original study [41]. Because the USER was administered to healthy participants that were not involved in a rehabilitation process, items of the domain restrictions were not collected. Items of the construct frequency were scored on a 6-point Likert scale with the scores 0, 20, 40, 60, 80 or 100. Items of the construct satisfaction were scored on a 5-point Likert scale with the scores 0, 25, 50, 75, or 100. Total scores on the different constructs were calculated by averaging the scores for the relevant items. Higher scores indicate higher levels (i.e. frequency or satisfaction) of participation in daily life.

### Procedure

In 2018, we distributed an online survey via Qualtrics software [42] that contained the questionnaires mentioned above. All participants were invited to take part on a voluntary basis. The majority of the sample (n = 2,032) were recruited via Panel Inzicht, a company specialized in online quantitative research where participants receive a financial reward for participation. In order to determine the test-retest reliability of the SVC, a subset (n = 190) of the total sample were recruited via other sources, including social media and via the distribution of flyers in university buildings, healthcare centers and supermarkets. These participants were invited to complete a subset of the questionnaires (i.e. SVC, BRIEF-A, SIMS) again after a period of two weeks. In case of no response after one week, a reminder was sent. The retest time interval of two weeks was based on quality criteria proposed by Terwee et al. [43], considering that the time period in-between assessments should be long enough to negate the effects of memory without allowing the condition to change. Time to complete all questionnaires was estimated to take around 40–50 minutes. Time to complete the re-assessment was around 10–15 minutes.

### Ethics statements

The Ethical Committee Psychology of the University of Groningen approved the study. All participants were informed about the study prior to study inclusion. Written informed consent was obtained from all initially included participants.

### Statistical analyses

SPSS Statistics version 23 [44] and LISREL 8.8 for Windows [45] were used for data analysis. Alpha level was set at p<0.05. Before running the analyses, we evaluated the outliers of the SVC by detecting and removing participants with repeating, inconsistent and improbable answer tendencies that were not detected by the BRIEF-A and the SIMS (e.g. repetition of the same answer categories over multiple questionnaires, a maximum score on multiple questionnaires, short administration time of 10 minutes or lower). Furthermore, assumptions of normality and linearity were checked.

#### Qualitative evaluation of the SVC

To evaluate whether the SVC is able to cover the most frequently reported visual complaints in the community sample, we qualitatively analyzed the answers on the first open-ended item of the SVC. Therefore, we first categorized the visual complaints participants reported. If there was any doubt about how to categorize certain complaints, this was discussed with collaborative authors to reach consensus. By the use of frequency analyses, we evaluated to what extent these categories of self-reported complaints covered the content of the structured 19 items of the SVC.

#### Factor structure

To evaluate the factor structure of the SVC, the data set was randomly split in two subsamples. For all other analyses the complete sample was used. Exploratory factor analysis was performed on the first subsample using principal axis factoring with oblique rotation. We performed parallel analysis to determine the number of factors to retain. In parallel analysis, random data-matrices of similar size as the actual data set were generated and eigenvalues were computed for the correlation matrices of each of the random data sets [46]. Subsequently, the eigenvalues of the random data sets were compared with the eigenvalues generated from the exploratory factor analysis. Factors were retained if the eigenvalues of the actual data set exceeded the eigenvalues of the random dataset. Additionally, inspection of the scree plot was performed to support the factor extraction criterion in parallel analysis. Subsequently, the proposed model from the exploratory factor analysis was validated by a confirmatory factor analysis on the second subsample. The Diagonally Weighted Least Square estimation method was applied in the confirmatory factor analysis because of the use of ordinal data. Scaling of the latent variables was achieved by limiting the variance of the factors to zero. The factor structure of the hypothesized model was examined by a number of goodness-of-fit statistics: the Chi-Square value, the normed Chi-Square value (χ^2^/df), the Root Mean Squared Error of Approximation (RMSEA), 90% confidence interval of the RMSEA, Standardized Root Mean Square Residual (SRMR), and the Comparative Fit Index (CFI). In addition, fit statistics of the hypothesized model were compared to the fit statistics of a competitive single-factor model to evaluate whether the hypothesized model proved a better fit to the data than a single-factor model.

The Chi-Square value derives from the comparison of the hypothesized model with a perfect fit of the data. The probability value associated with the Chi-Square value represents the likelihood of similarity between the hypothesized model and a perfect fit. A non-significant Chi-Square value indicates a good fit of the model. Disadvantages of the Chi-Square value are that deviations from normality and large sample sizes may result in model rejection [47]. This limitation can be addressed by computing a normed Chi-Square value, which can be calculated by dividing the Chi-Square value by the degrees of freedom (χ^2^/df). Less weight was therefore given to the Chi-Square value than to the normed Chi-Square value. The smaller the (normed) Chi-Square value, the better the fit of the model. Recommendations for an acceptable normed Chi-Square value range from 2.0 to 5.0 [48]; a value smaller than 3.0 reflects good model fit.

The RMSEA reflects the discrepancy of the hypothesized model compared to the population. The value is expressed per degree of freedom, and therefore takes the number of parameters estimated in the model into account. Smaller values indicate a better fit to the data, where values below 0.07 indicate a good fit [49]. LISREL provides a 90% interval around the RMSEA value, which reflects an estimate of (im)precision of the value and therefore assists in the evaluation of model fit. The upper limit of the confidence interval should be less than 0.08 in well-fitting models [47].

The SRMR represents the average value of the standardized residuals derived from the fitting of the hypothesized model to the sample data. The SRMR value ranges from 0 to 1. A value of 0.08 or smaller indicates a good fit of the model [50].

The CFI is a revised version of the Normed Fit Index and derives from the comparison of the hypothesized model with no existing model [51]. The difference to the Normed Fit Index is that the CFI takes sample size into account. Values of the CFI range from 0 to 1 with higher values indicating better fit of the model. A CFI equal to or higher than 0.90 indicates a good fit of the model [50].

#### Internal consistency

Internal consistency of the SVC and its factors was examined using Cronbach’s alpha. A Cronbach’s alpha of 0.70 and higher was considered as good internal consistency [43].

#### Convergent and divergent validity

Convergent and divergent validity were evaluated using non-parametric Spearman’s correlation analyses. For the evaluation of convergent validity, the SVC was correlated with the CVC-q and the NEI-VFQ-25. Divergent validity was evaluated by correlating the SVC with the BRIEF-A, FEDA, DASS-21 and the SIMS. We used Cohen’s criteria for the evaluation of convergent and divergent validity [52]. Following Cohen’s criteria, correlations of 0.10 are categorized as ‘low’, correlations of 0.30 as ‘medium’, and correlations of 0.5 and higher as ‘high’. A correlation of 0.50 or higher, comprising a shared variance of at least 25%, was used as an indication of sufficient convergent validity, where a correlation of 0.30 or lower, relating to a shared variance of maximally 9%, was used as an indication of sufficient divergent validity. Correlations of divergent validity were expected to be smaller than correlations of convergent validity.

#### Reproducibility

Test-retest reliability of the SVC and its factors was evaluated by calculating intraclass correlation coefficients (ICC; two-way random model, absolute agreement, single measures) between the two assessments. For the item of limitations in daily life, a weighted Cohen’s Kappa coefficient was calculated. An ICC or weighted Kappa of at least 0.70 is recommended as a minimum standard for acceptable reliability [43,53].

#### Participation in daily life

To evaluate the relation between subjective visual complaints and participation in daily life, the scores in the SVC were correlated with the scores in the USER questionnaire and with the SVC item that reflects patients’ self-rated limitations in daily life. Non-parametric Spearman’s correlation analyses were used. It was hypothesized that visual complaints were moderately related to a lower level of participation and a higher limitation in daily life.

Since floor effects of the SVC were expected in our community sample (i.e. total scores of 0 which represent no visual complaints), the analyses were repeated regarding convergent and divergent validity, internal consistency, test-retest reliability and participation in daily life for only the participants who reported visual complaints (total score>0).

## Results

### Participants

**Fig 1:**
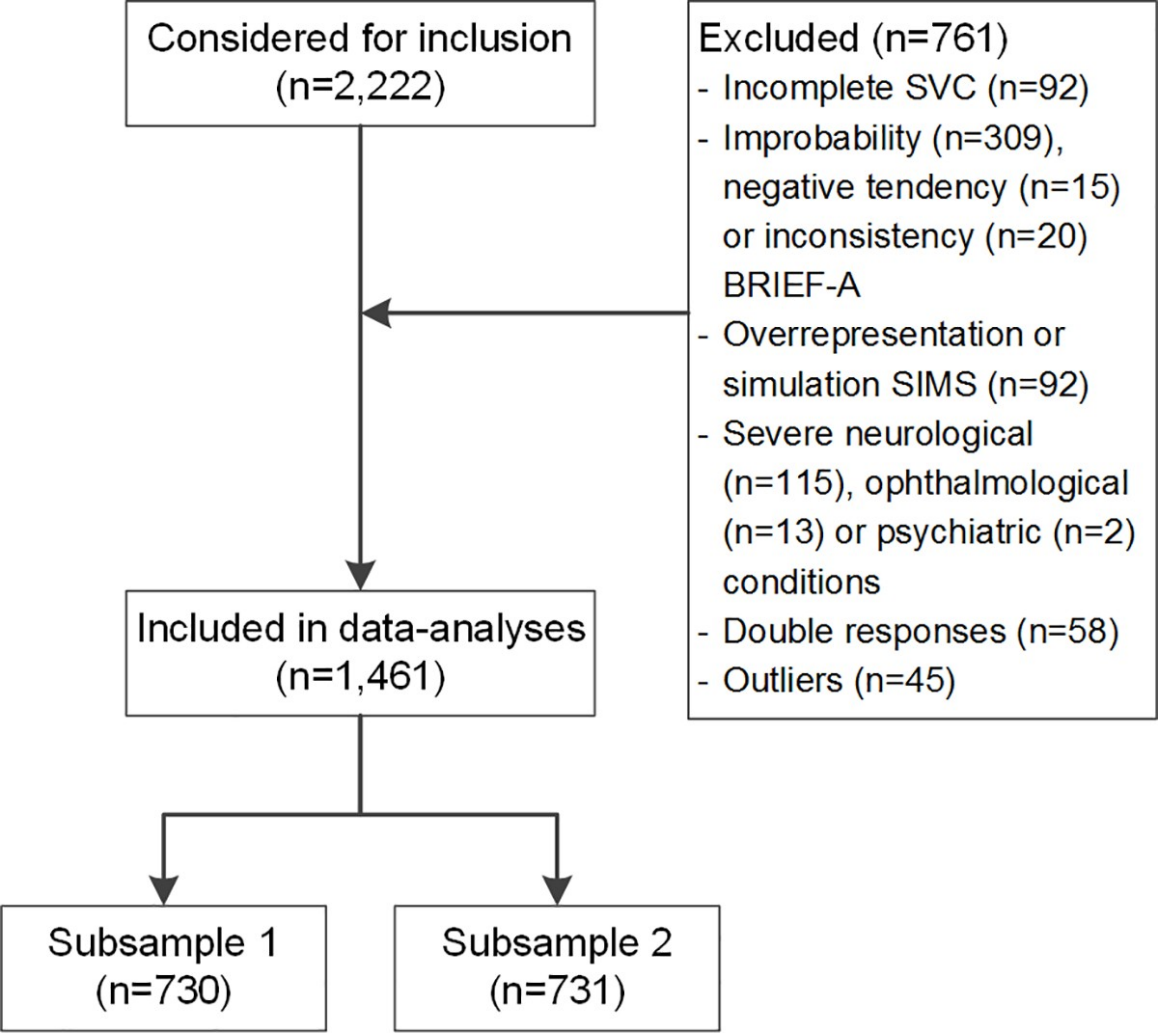
Flowchart of inclusion. SVC, Screening of Visual Complaints questionnaire; BRIEF-A, Behavior Rating Inventory of Executive Function-Adults; SIMS, Structured Inventory for Malingered Symptomatology.

A total of 2,222 participants were considered for inclusion (Fig 1). From this sample, 92 were excluded due to incomplete responses on the SVC. Furthermore, participants were excluded when the BRIEF-A revealed an improbability (n = 309), a negative tendency (n = 15) or an inconsistency (n = 20) of answers or when the SIMS indicated signs of overrepresentation or simulation of symptoms (n = 92). In total, 130 participants were excluded due to the presence or history of a severe neurological condition (n = 115), ophthalmological condition (n = 13) or psychiatric condition (n = 2). Finally, we removed double responses of the same participants (n = 58), and we removed those outliers of the SVC that showed repeating, improbable or inconsistent answer tendencies on the survey (n = 45). In total, data of 1,461 participants were used for analyses (Table 1). Participants’ age ranged from 18 to 95 years with a mean age of 54.9 years. To perform an exploratory and confirmatory factor analysis, the sample was randomly split into two subsamples. The two samples did not differ in age (t(1458) = 1.86, p = 0.064), sex (χ^2^(1) = 1.53, p = 0.217) or educational level (χ^2^(2) = 0.81, p = 0.668). The group of participants who were excluded (n = 703; we removed the 58 double responses from the total excluded group of 761 participants leaving 703 unique responses) were 2.9 years younger (52.0 versus 54.9, t(2156) = -3.3, p = 0.001, Hedges H = 0.15), had a lower education (low 22% versus 18%, medium 40% versus 43%, high 38% versus 39%, χ^2^(2) = 7.69, p = 0.021, Cramer’s V = 0.06), and consisted of less females (47% versus 54%, χ^2^(1) = 9.52, p = 0.002, Cramer’s V = 0.07) than the included sample.

**Table 1:** Characteristics of participants.

	Subsample 1	Subsample 2	Total sample
N	730	731	1,461
Age, years, mean (SD)	54.0 (18.9)	55.8 (18.3)	54.9 (18.6)
Sex, female, n (%)	404 (55%)	381 (52%)	785 (54%)
Educational level^a^, n (%)			
Low	124 (17%)	129 (18%)	253 (18%)
Medium	325 (45%)	308 (42%)	633 (43%)
High	280 (38%)	292 (40%)	572 (39%)

^a^ Based on the International Standard Classification of Education (ISCED), categorized into low, medium and high levels of education according to De Vent et al. [54]

### Self-reported visual complaints and limitations in daily life

On the first open-ended question of the SVC, 503 of the 1,461 participants (34%) reported to sometimes experience visual problems in daily life, while 101 participants (7%) reported to often experience visual problems in daily life. These 604 participants reported a total of 946 complaints. Of these complaints we disregarded reports that referred to short- or far-sightedness or to wearing glasses or contact lenses (n = 200), reports that were not described in terms of visual complaints (e.g. stomach problems, hay fever, headache, fatigue; n = 68) or not specific enough (e.g. low visibility; n = 44), and reports that referred to an ophthalmological condition (e.g. cataract, macular degeneration or amblyopia; n = 56), resulting in a total number of 578 visual complaints that were categorized (Table 2). These visual complaints were mostly related to unclear vision (29%) and trouble reading (23%). Of the 578 complaints being mentioned, 470 (81%) were covered by the structured items of the SVC. The remaining 19% of the complaints not being covered by the SVC were mostly related to seeing spots, floaters or opacities in the eyes (8%), tearing of the eyes (3%) and tiredness of the eyes (3%).

**Table 2:** Self-reported visual complaints on the first item of the SVC.

Self-reported visual complaints covered by the 19 structured items of the SVC			Self-reported visual complaints not being covered by the 19 structured items of the SVC		
	N	%		N	%
Unclear vision	169	29%	Spots, floaters or opacities in the eyes	48	8%
Trouble reading	134	23%	Tearing of the eyes	20	3%
More light needed	28	5%	Tiredness of the eyes	15	3%
Double vision	23	4%	Difficulty watching television or using the computer	9	2%
Trouble focusing	22	4%	Difficulty with gross motor activities	5	<1%
Painful eyes	21	4%	Difficulty with fine motor activities	3	<1%
Blinded by bright light	20	3%	Difficulty opening or closing eye lids	3	<1%
Dry eyes	18	3%	Sensitive or itching eyes	2	<1%
Vision problems in traffic	9	2%	Feelings of pressure behind the eyes	2	<1%
Problems with depth perception or estimating distances	8	1%	Problems recognizing people	1	<1%
Seeing shaky, jerky or shifting images	7	1%			
Seeing things that others do not	5	<1%			
Part(s) of the visual field missing	4	<1%			
Altered color experience	2	<1%			
**Total**	**470**	**81%**	**Total**	**108**	**19%**

### Exploratory factor analysis

An exploratory factor analysis was performed on the first subsample (n = 730) using principal axis factoring. A Kaiser-Meyer-Olkin value of 0.89 indicated excellent sampling adequacy and can be categorized as ‘marvelous’ according to Kaiser [55]. Results from the Bartlett’s Test of Sphericity indicated the data to be significantly different from an identity matrix (χ^2^(171) = 3347.8, p<0.001), meaning that correlations between items were sufficiently large for performing a factor analysis. Principal axis factoring using oblique rotation identified five factors meeting the criteria of an eigenvalue>1, explaining respectively 28.6, 7.7, 6.8, 5.7 and 5.5 percent of variance. Inspection of the scree-plot revealed a break after the first as well as after the third factor, which suggests extracting either one or three factors. Parallel analysis resulted in 3 factors to retain. We decided to retain three factors since the criteria resulting from parallel analysis are preferred and were supported by the scree-plot [46]. Table 3 shows the results of the principal axis factoring with the rotated factor loadings of each of the items and the eigenvalues and explained variance of each of the three factors. The three factors were interpreted as follows: *diminished visual perception* (factor 1; 11 items), *altered visual perception* (factor 2; 6 items), and *ocular discomfort* (factor 3; 2 items).

**Table 3:** Results of the principal axis factoring.

	Factor loadings		
Item	Factor 1	Factor 2	Factor 3
Item 2. Unclear vision	**.778**	-.064	-.035
Item 3. Trouble focusing	**.642**	.022	.081
Item 9. Trouble seeing at reduced contrast	**.622**	-.018	.010
Item 20. Trouble reading	**.612**	.001	-.173
Item 17. More time needed	**.564**	.067	.094
Item 11. More light needed	**.505**	.043	-.036
Item 12. Difficulty adjusting to light or dark	**.442**	-.044	.167
Item 10. Blinded by bright light	**.420**	.056	.205
Item 18. Vision problems in traffic	**.371**	.199	.067
Item 19. Trouble looking for objects	**.369**	.160	.035
Item 5. Problems with depth perception or estimating distances	**.301**	.168	.135
Item 14. Altered perception of objects or faces	.114	**.556**	-.065
Item 13. Seeing things that others do not	-.055	**.536**	.070
Item 6. Seeing shaky, jerky or shifting images	-.031	**.521**	.143
Item 4. Double vision	.011	**.495**	-.020
Item 7. Missing part(s) of the visual field	.110	**.472**	-.081
Item 8. Altered color experience	-.009	**.471**	-.021
Item 16. Dry eyes	.092	-.040	**.608**
Item 15. Painful eyes	.009	.099	**.557**
**Eigenvalue**	**5.44**	**1.45**	**1.29**
**Explained variance**	**28.61%**	**7.66%**	**6.78%**

Factor loadings in bold represent items’ highest factor loadings

### Confirmatory factor analysis

A confirmatory factor analysis was performed on the second subsample consisting of 731 participants, which exceeded the criterion of a minimum sample size of 200 respondents for confirmatory factor analysis as proposed by Hinkin [48]. The Chi-Square value of the hypothesized three-factor model indicated the model to be significantly different from the sample data (χ^2^(149) = 313.99, p<0.01). However, more importantly, the normed Chi-Square value (χ^2^/df) of 2.11 revealed an acceptable fit of the model. The RMSEA value of 0.039 (CI = 0.033–0.045) was below 0.070, and the upper limit of the CI was below 0.080, further supporting model fit. In addition, the CFI value of 0.99 and the SRMR value of 0.068 further supported model fit. In summary, goodness of fit statistics of the confirmatory factor analysis revealed a satisfactory fit of the hypothesized three-factor model. Although the single-factor model showed satisfactory fit as well (χ^2^(152) = 351.07, p<0.05; χ^2^/df = 2.31; RMSEA [CI] = 0.042 [0.037–0.048]; CFI = 0.99, SRMR = 0.076), the three-factor model outperformed the single-factor model in all of the goodness-of-fit statistics, with the exception of the CFI value.

### Internal consistency

A Cronbach’s alpha of 0.85 (CI = 0.84–0.86) revealed high internal consistency of the total questionnaire. In addition, the internal consistency of factor 1 (Cronbach’s α = 0.84, CI = 0.82–0.85) was high. Cronbach’s alphas of factor 2 (Cronbach’s α = 0.62, CI = 0.59–0.65) and factor 3 (Cronbach’s α = 0.49, CI = 0.44–0.54) were below 0.70 and therefore considered as insufficient. Deleting items did not increase the internal consistency of the total questionnaire nor the factors.

### Convergent validity

Table 4 shows the correlations of the SVC with the CVC-q and with the NEI-VFQ-25. All correlations were found significant with p<0.001, with the exception of the correlation between the NEI-VFQ-25 subscale color vision and the factor *ocular discomfort* with p = 0.006 (Table 4). The SVC showed strong correlations with the NEI-VFQ-25 (r = -0.71) and with the CVC-q total score (r = 0.84). In addition, strong correlations were found between the SVC and NEI-VFQ-25 subscales general vision (r = -0.55), near activities (r = -0.55), distance activities (r = -0.55) and mental health (r = -0.56). The SVC correlated weakly with the NEI-VFQ-25 subscales color vision (r = -0.21) and dependency (r = -0.28).

**Table 4:** Convergent validity of the SVC with the CVC-q and the NEI-VFQ-25.

	SVC total score	1. Diminished visual perception	2. Altered visual perception	3. Ocular discomfort
CVC-q—total score	0.84*	0.84*	0.51*	0.31
NEI-VFQ-25^a^ –total score	-0.71*	-0.68*	-0.41	-0.39
General health^b^	-0.33	-0.31	-0.24	-0.17
General vision^b^	-0.55*	-0.55*	-0.37	-0.22
Near activities	-0.55*	-0.57*	-0.31	-0.20
Distance activities	-0.55*	-0.54*	-0.35	-0.27
Driving	-0.47	-0.47	-0.23	-0.21
Peripheral vision^c^	-0.40	-0.38	-0.32	-0.20
Color vision^c^	-0.21	-0.20	-0.20	-0.07
Ocular pain^b^	-0.46	-0.38	-0.27	-0.57*
Role difficulties	-0.49	-0.49	-0.30	-0.20
Dependency	-0.28	-0.27	-0.25	-0.16
Social functioning	-0.31	-0.30	-0.31	-0.12
Mental health	-0.56*	-0.55*	-0.36	-0.25

SVC, Screening of Visual Complaints questionnaire; CVC-q, Cerebral Visual Complaints questionnaire; NEI-VFQ-25, National Eye Institute Visual Function Questionnaire-25

^a^ In contrast to the SVC and the CVC-q, a higher score on the NEI-VFQ-25 questionnaire indicates better visual function

^b^ Subscale composed of two items

^c^ Subscale composed of a single item

* Correlations of high strength according to Cohen’s criteria

The factor *diminished visual perception* of the SVC showed strong correlations with the CVC-q and NEI-VFQ-25 (r = 0.84; r = -0.68, respectively); the factor *altered visual perception* of the SVC showed moderate to strong correlations (r = 0.51; r = -0.41, respectively), and the factor *ocular discomfort* of the SVC showed correlations of moderate strength (r = 0.31; r = -0.39, respectively). Correlations between the factors of the SVC and the subscales of the NEI-VFQ-25 ranged from weak to strong. Strong correlations were observed between the factor *ocular discomfort* and the NEI-VFQ-25 subscale ocular pain (r = -0.57) and between the factor *diminished visual perception* and the NEI-VFQ-25 subscales general vision (r = -0.55), near activities (r = -0.57), distance activities (r = -0.54) and mental health (r = -0.55).

### Divergent validity

Table 5 shows the correlations regarding divergent validity of the SVC with the BRIEF-A, FEDA, DASS-21 and the SIMS. All correlations were found significant with p<0.001. The SVC correlated weakly with the BRIEF-A (r = 0.29) and the SIMS (r = 0.26) but showed moderate correlations with the FEDA (r = 0.40), the DASS-21 (r = 0.34) and the anxiety subscale of the DASS-21 (r = 0.34). The factors of the SVC showed weak correlations with all of the divergent measures, with the exception of the factor *diminished visual perception*, which showed correlations of moderate strength with the FEDA (r = 0.37) and DASS-21 (r = 0.30).

**Table 5:** Divergent validity of the SVC with the BRIEF-A, the FEDA, the DASS-21 and the SIMS.

	SVC—total score	1. Diminished visual perception	2. Altered visual perception	3. Ocular discomfort
BRIEF-A–total score	0.29^#^	0.26^#^	0.23^#^	0.13^#^
Metacognition	0.27^#^	0.25^#^	0.21^#^	0.13^#^
Behavioral regulation	0.26^#^	0.24^#^	0.22^#^	0.19^#^
FEDA	0.40	0.37	0.29^#^	0.19^#^
DASS-21 –total score	0.34	0.30	0.27^#^	0.20^#^
Depression	0.25^#^	0.23^#^	0.22^#^	0.12^#^
Anxiety	0.34	0.29^#^	0.28^#^	0.26^#^
Stress	0.27^#^	0.24^#^	0.22^#^	0.14^#^
SIMS	0.26^#^	0.23^#^	0.21^#^	0.20^#^

SVC, Screening of Visual Complaints questionnaire; BRIEF-A, Behavior Rating Inventory of Executive Function-Adults; FEDA, Questionnaire for Experiences of Attention Deficits (*Fragebogen erlebter Defizite der Aufmerkzamkeit*); DASS-21, Depression Anxiety Stress Scale–21; SIMS, Structured Inventory for Malingered Symptomatology

^#^ Correlations of low strength according to Cohen’s criteria

### Reproducibility

Of the 190 participants who have been invited for reassessment of the SVC, 85 participants completed the second assessment. From these participants, 19 were excluded from the analyses because of the following reasons: an improbability (n = 12), a negative tendency (n = 2), or an inconsistency (n = 1) of answers on the BRIEF-A in either the first or second assessment; signs of overrepresentation or simulation of symptoms on the SIMS (n = 1); the presence of a neurologic condition (n = 1). Finally, we removed those outliers of the SVC which showed improbable or inconsistent answer tendencies on the survey (n = 2). In total, 66 participants were included for test-retest analysis. A sample size of at least 50 is considered adequate to assess a measure of agreement according to Altman [56].

All participants completed the reassessment between 14 and 38 days, with exception of one participant who completed the reassessment in 69 days. The median retest period was 15 days. The subgroup of participants included for the test-retest analysis were 6.8 years younger (M = 48.4 versus 55.2, t(73) = 3.2, p = 0.002, Hedges’ g = 0.37), had a higher education (low 0% versus 18%, medium 33% versus 44%, high 67% versus 38%, (χ^2^(2) = 26.88, p = <0.001, Cramer’s V = 0.14) and included more females (76% versus 53%, χ^2^(1) = 13.49, p = <0.001, Cramer’s V = 0.10) than the sample who did not complete the re-assessment (n = 1,395).

The SVC showed an ICC of 0.82 (CI = 0.72–0.88), indicating acceptable test-retest reliability of the total questionnaire. Sufficient test-retest reliability was also found for the factor *diminished visual perception* (ICC = 0.84, CI = 0.75–0.90) and the factor *altered visual perception* (ICC = 0.74, CI = 0.60–0.84). However, the test-retest reliability of the factor *ocular discomfort* was below 0.70 and was therefore considered as insufficient (ICC = 0.64, CI = 0.47–0.76). The weighted Cohen’s Kappa of the item of limitations in daily life was 0.67.

### Participation in daily life

Participants reported a median limitation in daily life due to visual complaints of 2 (IQR = 1–4) on a scale from 0 (no limitations) to 10 (very severe limitations). This item of reported limitations in daily life correlated with the SVC total score (r = 0.68, p<0.001). The SVC total score showed a significant but weak correlation with the subscale satisfaction of the USER questionnaire (r = -0.18, p<0.001), whereas a non-significant correlation was found with the subscale frequency (r = -0.01, p = 0.763).

### Repeating analyses without floor scores

The analyses of convergent and divergent validity, internal consistency, test-retest reliability and the relation with participation in daily life were repeated on the dataset without participants who reported no visual complaints on the SVC (i.e. total scores of 0; n = 199). Repeating the analyses on this sample did not change the interpretation of results (S3 Appendix).

## Discussion

The newly developed SVC aims to screen for visual complaints in patients with neurodegenerative disorders, including PD, MS or dementia, in order to improve referral to clinical care, assessment and rehabilitation. As a first step, this study assessed the psychometric properties of the SVC in a large community sample, by examining its factor structure, convergent and divergent validity, internal consistency, and test-retest reliability. Furthermore, we aimed to evaluate the relation between visual complaints and participation in daily life.

### Factor structure

Three factors were extracted from the SVC, summarized as *diminished visual perception*, (11 items), *altered visual perception* (6 items) and *ocular discomfort* (2 items). The first factor *diminished visual perception* explained 28% of variance in items scores. It comprises complaints related to visual function in visual acuity and light disturbances (e.g. unclear vision, being blinded by bright light, having trouble seeing at reduced contrast and experiencing difficulty adjusting to light or dark) as well as complaints related to daily-life activities (e.g. having trouble reading, experiencing vision problems in traffic and having trouble looking for objects). This shared factor of complaints related to visual function and daily-life activities might indicate that these complaints are interrelated. Comparable findings were seen in a study of Raphael and colleagues [57], in which the factor structure of a 10-item supplemental questionnaire, developed to increase the capacity of the NEI-VFQ-25, was evaluated. Exploratory factor analyses of this supplement revealed two distinct factors: one large factor of 8 items and a small factor of 2 items. Just as in the current study, the first factor consisted of items related to complaints about visual function (e.g. ocular tiredness, glare sensitivity, different binocular eyesight, blurry vision and diplopia) and daily-life activities (e.g. parking a car and using a computer) [57]. These previous findings support the interrelation between complaints in visual function and daily-life activities and suggests that individuals who have complaints in visual function are likely to exhibit difficulties in performing daily-life activities, and vice versa.

The second factor, labeled as *altered visual perception*, explains 7% of variance in item scores. It is composed of 6 items measuring complaints of alterations in visual perception, including double vision, an altered perception of objects or faces, an altered color experience, seeing things that others do not see, missing part(s) of the visual field and seeing shaky, jerky or shifting images. The third factor, explaining 6% of the variance in item scores, is labeled as *ocular discomfort* and consists of 2 items measuring complaints of dry and painful eyes. A similar two-item construct of ocular pain can be found in the NEI-VFQ-25. Although seen in several health-related questionnaires, it can be questioned whether a two-item construct is clinically meaningful to extract [30,57]. In the study of Raphael and colleagues [57] also a two-item construct, related to eye/lid appearance, was extracted. The authors concluded that the two items within this factor may be distinct from the items of the 8-item factor of visual dysfunction. Likewise, in the present study, the two items related to *ocular discomfort* appear to be distinct from the items that assess *diminished visual perception* or *altered visual perception*, since the two-item construct of *ocular discomfort* explained a sufficient amount of variance not already covered by these large multiple-item factors. The factor *ocular discomfort* has therefore statistically proven its value in assessing a distinct significant construct. The remaining question is whether the factor would also be of clinical value. Possibly complaints related to ocular discomfort would be a reason to think of functional or anatomical damage to the eye. If that is the case, referral to an ophthalmologist would be more suitable than referral to specialized visual rehabilitation centers for visual care and rehabilitation. Further research in clinical populations should be aimed at evaluating the clinical value of this construct in the referral process.

The three-factor model showed good model fit, and even outperformed a single-factor model. It therefore can be concluded that this three-factor model underlies the SVC and that this model is valid in a community sample. However, the single-factor also proved good fit to the data, where it could be discussed whether to just stick to a single-factor model as well. Also, the dominance of the first factor, which explained 28% of the variance, may support a single-factor model instead of a multiple-factor model. Simple models are generally preferred above complex models in explaining item scores, provided that the more complex model does not significantly explain more variance in items scores. Different studies therefore decided to retain a single-factor structure instead of a multiple-factor structure [58,59]. A disadvantage of using a single score, however, is that it could be associated with loss of information (i.e. explained variance) in construct scores. The question remains whether the three-factor model significantly explains more variance in item scores compared to the single-factor model, or whether the added value of the three-factor model is negligible. Further evaluation of the factor structure of the SVC in a clinical sample is necessary in order to examine whether a three-factor model or a single-factor model holds in clinical samples.

### Convergent and divergent validity

In line with expectations, the SVC showed strong correlations with the visual questionnaires CVC-q and NEI-VFQ-25 and the NEI-VFQ-25 subscales general vision, near activities and distance activities, supporting convergent validity of the SVC. In addition, the factors of the SVC correlated moderately to strongly with the CVC-q and the NEI-VFQ-25, except for the factor *ocular discomfort*, which showed only moderate correlations. Remarkable, however, is that the factor *ocular discomfort* did strongly correlate with the subscale ocular pain of the NEI-VFQ-25, supporting convergent validity of this factor as well. It has to be pointed out that the SVC shares half of its items with the CVC-q because the two final questionnaires were developed parallel to each other. This, of course, adds to the high correlation between these two measures. However, correlating the SVC score with the CVC-q total score without the overlapping items still resulted in a strong correlation (r = 0.67), providing further support for sufficient convergent validity.

Regarding divergent validity, we hypothesized that the construct of visual complaints measured with the SVC would be different from the constructs of self-reported executive function, attentional function, symptoms of depression, anxiety and stress, and symptom validity. As expected, weak correlations were found between the SVC and symptom validity (SIMS) and executive function (BRIEF-A). In contrast, moderate correlations were found with attentional function (FEDA) and symptoms of depression, anxiety and stress (DASS-21 total score), the latter in particular with the subscale anxiety. These results suggest that having visual complaints relates to feelings of anxiety and worries or fear, which is consistent with the strong correlation that was found between the SVC and the mental health subscale of the NEI-VFQ-25, which consists of items related to worries about one’s eyesight. It seems plausible that individuals who have visual complaints experience more feelings of anxiety due to these complaints. The same may apply for attentional deficits, of which a moderate correlation was found with the SVC. Individuals who experience visual complaints might experience more trouble with keeping their attention when carrying out different kinds of activities which require visual function. This argument is also in line with the finding of a composite factor of items related to primary visual function and performing visual daily-life activities, found in the present study (i.e. diminished visual perception) and in the study of Raphael and colleagues [57]. In summary, the correlations regarding divergent validity were found to be smaller than correlations regarding convergent validity, which was in line with our expectations. The SVC shared 50 to 71% of its variance with comparable visual questionnaires and shared only 7 to 16% of its variance with the divergent measures. Thus, even though results of divergent validity were not totally conform to expectations, overall the results clearly indicate sufficient construct validity of the SVC.

### Reliability

The SVC questionnaire showed good internal consistency and test-retest reliability. With regard to the individual factors, the factor *diminished visual perception* was found reliable regarding internal consistency and test-retest reliability, the factor *altered visual perception* showed sufficient test-retest reliability but insufficient internal consistency, and the factor *ocular discomfort* showed insufficient internal consistency as well as test-retest reliability. For the factor *ocular discomfort*, it should be noted that this construct consists of only two items and Cronbach’s alpha is highly dependent on the number of items included [43]. Comparable results were found in the study of Mangione et al. [31], where the two-item subscale expectations of the NEI-VFQ was of similar insufficient internal consistency. Future research should be aimed at evaluating reliability measures of the SVC in clinical populations of patients with neurodegenerative disorders. In particular, test-retest reliability measures in these clinical populations are desirable, since clinicians may want to assess the SVC multiple times in the same patients to screen for visual complaints that may not have been present before or to screen for any change in visual complaints over time.

### Participation in daily life

A high correlation was found between the SVC and self-rated limitations in daily life due to visual complaints. These results indicate that individuals with visual complaints feel limited in daily life due to these complaints, emphasizing the need of visual care and rehabilitation for people experiencing visual complaints. The SVC, however, did not or only weakly correlate with the subscales frequency and satisfaction of the USER questionnaire, respectively. This indicates that no clear relation exists between the presence of visual complaints and the frequency and satisfaction of participation in daily life (e.g. work, education, house-holding activities, sports, leisure activities or visiting family and friends). These results seem to be in contrast with the argument that visual complaints and daily-life functioning are interrelated, resulting from the shared factor of visual complaints and daily-life activities found in the present study (i.e. *diminished visual perception*) and in the study of Raphael and colleagues [57]. An explanation might be that the SVC focuses on daily-life functioning in relation to vision where the USER focuses on participation in daily life not specifically related to vision. In other words, visual complaints may be related to functioning or participation in daily-life, but only in those activities where visual function is of relevance. Furthermore, these results apply to the general population; results may be different in a sample of patients with visual impairments.

### Clinical implications

Based on the abovementioned results, we recommend the SVC to be used by medical specialists, including general practitioners and neurologists, in order to optimize referral of visual impaired patients to specialized care and treatment. Because of its short length the SVC is quick and easy in use, increasing clinical efficiency. The SVC can be of great value compared to the existing visual-health questionnaires, such as the NEI-VFQ, which are generally long and do not always focus on subjective visual complaints at a functional level. In addition, the SVC includes items that are often reported by patients with PD or MS, such as different perception of objects or faces, seeing things that others do not see, double vision, being blinded by bright light, seeing shaky, jerky or shifting images and missing part(s) of the visual field, which are not all being captured by existing visual questionnaires such as the CVC-q and the NEI-VFQ-25. These specific items may be important contributors in detecting visual complaints in these patient groups. Besides its purpose for patients with neurodegenerative disorders, the SVC may be suitable for the assessment of visual complaints in other patient groups as well, for example in patients with other etiologies of acquired brain injury.

When using the SVC in clinical practice, the SVC can be best interpreted by the defined factor scores and by a general total score. Scores on the factors can be calculated to give a reliable indication of the defined construct and, in the case of repeated administrations, to measure change over time Since not all factors show sufficient internal consistency or reliability (except for the factor *diminished visual perception*), we do recommend caution in the interpretation of solely these factor scores and we recommend the individual items to be qualitatively analyzed as well. A composite score of the total SVC questionnaire can be useful to screen for the degree of reported visual complaints in general and to define overall change in visual complaints in the case of repeated administrations. High internal consistency and test-retest reliability of the total questionnaire support the use of this composite score.

Next to calculating scores, items should be qualitatively analyzed to serve as guidelines for patients’ individual assessment, priorities and needs. In particular, clinicians may want to pay attention to the first open-ended question, in which patients can report spontaneous complaints. Qualitative analysis of the SVC shows that the structured items cover approximately 80% of the complaints reported in the community sample, leaving 20% uncovered. This shows that people might have additional visual complaints apart from the ones being asked by the structured items. In this community sample, spontaneous complaints not being detected by the structured items of the SVC mostly refer to seeing spots, floaters or opacities, tearing of the eyes and tiredness of the eyes. It could be discussed whether to add items to the SVC covering these complaints as well. It should be noted, however, that these results apply to a community sample and cannot merely be generalized to patient samples. Administration of the SVC in clinical populations of patients with neurodegenerative disorders is needed in order to evaluate whether the SVC covers most of the complaints experienced by these patients or whether certain items might be missing or are possibly redundant.

### Limitations and directions for future research

The questionnaires in the present study were administered by means of self-report, while some of these questionnaires (e.g. CVC-q, NEI-VFQ-25) have initially been developed for interviewer administration and have not yet been validated in self-report administration. Changing the form of administration could yield different results. Self-administration, for example, has been shown to generate worse scores in vision-related quality of life than administration by telephone [60]. The same could apply in the present study: using a different form of administration might have interfered with the answers given on the questionnaires. However, since we used the same administration type for all questionnaires, we do not think that this has changed the results of construct validity.

A second limitation is that the present study, for the purpose of evaluating the validity of the SVC, solely assessed self-reported complaints of visual function and did not include objective measurements. We have chosen for self-report instruments of visual function for measuring convergent validity since we aimed for similar constructs, and questioned whether objective measures would resemble a similar construct as subjective complaints. Margolis and colleagues (2002) revealed that studies using self-report instruments to assess convergent validity of visual questionnaires generally resulted in moderate to large correlations, whereas correlations in studies assessing convergent validity with objectified measures (e.g. visual acuity) were generally small. Other studies aiming to evaluate the association between subjective and objective cognitive complaints also found small correlations [15–17]. Margolis and colleagues [14] therefore recommended subjective measures for evaluating the validity of visual health-related questionnaires, since the construct of subjective complaints and objective measures seems to be different. But more importantly, from their findings they concluded that subjective visual assessments provide additional insight into patients’ visual functioning that is complementary to objective clinical measures [14]. This underlines the importance of the application of such questionnaires in clinical practice. Future research should be aimed at further specifying the relationship between subjective and objective assessments of visual function.

A final limitation is that the current study is conducted in healthy participants from a community sample. Even though we aimed for an equal representation of age, gender and educational level in the included sample, we were not fully able to prevent group differences, as the excluded participants as well as the subgroup who was re-tested with the SVC slightly differed in age, gender, and educational level from the total included group. We, however, do not think that these differences altered the results since effect sizes were small to negligible. But, more importantly, results of this study only apply to healthy participants and therefore cannot merely be generalized to clinical samples of patients with neurodegenerative disorders. This means that we cannot assure that the SVC shows similar validity and reliability in these patient groups. Moreover, we do not know whether the SVC has the potential to recognize or capture the visual complaints these patients encounter in daily life, and in what way patients having visual complaints may differ from patients not having visual complaints. Future research should be aimed at evaluating these quality measures of the SVC in different clinical samples, such as patients with neurodegenerative disorders, including PD, MS or dementia, or patients with other etiologies of acquired brain injury. Furthermore, it would be valuable to evaluate how the SVC could be used in clinical decision making, by calculating cut-off scores for referral.

## Conclusions

The current study demonstrated that the SVC, composed of a three-factor structure, is a valid and reliable tool for the assessment of subjective visual complaints in a community sample. Therefore, the SVC seems a promising tool for use in clinical practice in patients with neurodegenerative disorders. We recommend medical specialists, including general practitioners and neurologists, to use the SVC in their clinical practice in order to optimize referral of visual impaired patients to specialized care and treatment.

## Supporting information

S1 Appendix The screening of visual complaints questionnaire [original Dutch questionnaire]. (PDF)

S2 Appendix The screening of visual complaints questionnaire [English translation]. (PDF)

S3 Appendix Results of convergent validity, divergent validity, test-retest reliability and the relation with participation in daily life for the data without SVC zero scores. (PDF)

S4 Appendix Checklist of the STARD guidelines. (DOCX)
