# Supplementary material for: The development of the Screening of Visual Complaints questionnaire for patients with neurodegenerative disorders: Evaluation of psychometric features in a community sample
Source: PLoS One. 2020 Apr 29;15(4):e0232232. doi: 10.1371/journal.pone.0232232 (PMC7190154; doi:10.1371/journal.pone.0232232)
Supplement: S1 Appendix — (PDF) [file pone.0232232.s001.pdf]

Screening Visuele Klachten (SVK)

Datum: ____________________

Naam: ____________________

Geslacht: ____________________

Geboortedatum: ____________________

Wat is uw hoogst afgeronde opleiding? ____________________

Dit is een vragenlijst met uitspraken over problemen die met uw zicht te maken hebben. Als u een bril of contactlenzen heeft, ga er dan bij de beantwoording van de vragen vanuit dat u deze draagt.
Elke vraag heeft meerdere antwoordmogelijkheden. Kies het antwoord dat het meest op u van toepassing is. Het gaat daarbij steeds om de <u>afgelopen weken</u>.
Als u niet zeker weet welk antwoord u moet kiezen, geef dan het best passende antwoord.
Kruis bij alle volgende vragen s.v.p. 1 antwoord aan. Er zijn in totaal 3 pagina's.

		**Ja**	**Nee**
	Bent u bekend bij een oogarts?	❒	❒
	Indien 'Ja':<br>Bij welke oogarts (of welk ziekenhuis) bent u bekend?		
	Voor welke oogheelkundige aandoening(en) bent u bekend bij de oogarts?		

		**Nee/ nauwelijks**	**Soms**	**Vaak/ altijd**
1	Ervaart u in het dagelijks leven problemen met uw zicht?	❒	❒	❒
	Indien 'Soms' of 'Vaak/altijd': Kunt u aangeven welke problemen of klachten u heeft met uw zicht?			
	a.			
	b.			
	c.			
	d.			

Huizinga F, Heutink J, de Haan GA, van der Lijn I, van der Feen FE, Vrijling ACL, Melis-Dankers BJM, de Vries SM, Tucha O, Koerts J. The development of the Screening of Visual Complaints questionnaire for patients with neurodegenerative disorders: evaluation of psychometric features in a community sample. PloS One. 2020

		Nee/ nauwelijks	Soms	Vaak/ altijd
2	Heeft u de indruk dat u minder scherp bent gaan zien?	❒	❒	❒
3	Heeft u moeite met scherpstellen of duurt het langer voordat u een scherp beeld heeft?	❒	❒	❒
4	Heeft u last van dubbelzien of dubbelbeelden?	❒	❒	❒
5	Heeft u moeite met dieptezien of afstanden inschatten?	❒	❒	❒
6	Heeft u last van trillende, schokkerige of bewegende beelden?	❒	❒	❒
7	Heeft u het idee dat u delen mist in het gezichtsveld?	❒	❒	❒
8	Ervaart u kleuren anders dan vroeger?	❒	❒	❒
9	Heeft u moeite met het zien bij verminderd contrast (bijv. wanneer letters niet zijn afgedrukt op een witte, maar op een grijze achtergrond)?	❒	❒	❒
10	Wordt u, meer dan vroeger, verblind door fel licht?	❒	❒	❒
11	Heeft u de indruk dat alles donkerder lijkt of heeft u meer behoefte aan licht dan vroeger?	❒	❒	❒
12	Heeft u moeite met het wennen aan licht of donker?	❒	❒	❒
13	Ziet u wel eens dingen die anderen niet zien (denk bijv. aan flitsen, patronen, voorwerpen of dieren)?	❒	❒	❒
14	Heeft u de indruk dat u voorwerpen of gezichten anders waarneemt, bijvoorbeeld vervormd of met nabeelden?	❒	❒	❒
15	Heeft u pijn aan uw ogen?	❒	❒	❒

Huizinga F, Heutink J, de Haan GA, van der Lijn I, van der Feen FE, Vrijling ACL, Melis-Dankers BJM, de Vries SM, Tucha O, Koerts J. The development of the Screening of Visual Complaints questionnaire for patients with neurodegenerative disorders: evaluation of psychometric features in a community sample. PloS One. 2020

	Nee/ nauwelijks	Soms	Vaak/ altijd
16 Heeft u last van droge ogen?	❐	❐	❐
17 Heeft u het idee dat u meer tijd nodig hebt om dingen te zien?	❐	❐	❐
18 Heeft u moeite met zien of waarnemen bij deelname aan het verkeer (lopen, fietsen en autorijden)?	❐	❐	❐
19 Heeft u, vanwege uw zicht, moeite met het zoeken en vinden van dingen?	❐	❐	❐
20 Heeft u, vanwege uw zicht, moeite met lezen?	❐	❐	❐

	**Geef een cijfer van 0 tot 10** *(omcirkel het juiste antwoord)*
21 In hoeverre wordt u in het dagelijks leven gehinderd door bovenstaande klachten met betrekking tot het zien?<br><br>*0 = geen hinder*<br>*10 = zeer ernstige hinder*	0 1 2 3 4 5 6 7 8 9 10

	**Ja**	**Nee**
Stelt u advies, onderzoek en/of revalidatie voor de hierboven genoemde klachten op prijs?	❐	❐

Wilt u controleren of u alle vragen heeft beantwoord?
Bij elke vraag dient 1 antwoord aangekruist te zijn.

Dank u wel. Dit is het einde van de vragenlijst.

Huizinga F, Heutink J, de Haan GA, van der Lijn I, van der Feen FE, Vrijling ACL, Melis-Dankers BJM, de Vries SM, Tucha O, Koerts J. The development of the Screening of Visual Complaints questionnaire for patients with neurodegenerative disorders: evaluation of psychometric features in a community sample. PloS One. 2020
