# Supplementary material for: The development of the Screening of Visual Complaints questionnaire for patients with neurodegenerative disorders: Evaluation of psychometric features in a community sample
Source: PLoS One. 2020 Apr 29;15(4):e0232232. doi: 10.1371/journal.pone.0232232 (PMC7190154; doi:10.1371/journal.pone.0232232)
Supplement: S2 Appendix — (PDF) [file pone.0232232.s002.pdf]

university of groningen

Visio

SCREENING VISUAL COMPLAINTS (SVC)

DISCLAIMER: The following questionnaire is a first English translation of the original Dutch questionnaire. This English version has not been validated yet and therefore does not guarantee similar validity and reliability as the Dutch version.

Date: ____________________

Name: ____________________

Sex: ____________________

Date of birth: ____________________

What is your highest level of education? ____________________

The following list of questions concerns problems that you may have with your eyesight.
If you wear glasses or contact lenses, please assume that you are wearing these when you answer the questions.
Each question has several possible answers. Please choose the answer that is most appropriate to your situation as it has been <u>over the past weeks</u>.
If you are not certain, please choose the answer that best reflects your situation.
Please choose 1 answer for each of the following questions. The questionnaire consists of 3 pages.

		**Yes**	**No**
	Did you ever visit an ophthalmologist?	❒	❒
	If 'Yes':<br>Which ophthalmologist did you visit or at which hospital did you visit an ophthalmologist?		
	For which ophthalmologic condition did you visit an ophthalmologist?		

		**No/ Hardly ever**	**Sometimes**	**Often/ Always**
1	Do you experience problems with your eyesight in daily life?	❒	❒	❒
	If 'Sometimes' or 'Often/Always': please describe your problems or complaints regarding your eyesight			
	a.			
	b.			

	c.			
	d.			
		**No/ Hardly ever**	**Sometimes**	**Often/ Always**
2	Do you have the impression that your vision has become less clear?	❐	❐	❐
3	Do you have trouble focusing or does it take longer before things are in focus?	❐	❐	❐
4	Do you have double vision or see double images?	❐	❐	❐
5	Do you have problems with depth perception or estimating distances?	❐	❐	❐
6	Do you see shaky, jerky or shifting images?	❐	❐	❐
7	Do you have the impression that you cannot see part(s) of the visual field?	❐	❐	❐
8	Do you experience colour differently than before?	❐	❐	❐
9	Do you have trouble seeing things at reduced contrast (e.g. letters that have not been printed on a white but on a grey background)?	❐	❐	❐
10	Are you more easily blinded by bright light than before?	❐	❐	❐
11	Do you have the impression that everything looks darker or that you need more light than before?	❐	❐	❐
12	Do you have difficulty adjusting to light or dark environments?	❐	❐	❐

Huizinga F, Heutink J, de Haan GA, van der Lijn I, van der Feen FE, Vrijling ACL, Melis-Dankers BJM, de Vries SM, Tucha O, Koerts J. The development of the Screening of Visual Complaints questionnaire for patients with neurodegenerative disorders: evaluation of psychometric features in a community sample. PloS One. 2020

13	Do you see things that others do not see (e.g. flashes of light, patterns, objects or animals)?	❒	❒	❒
14	Do you have the impression that you perceive objects or faces differently, for example, distorted or with afterimages?	❒	❒	❒
15	Are your eyes painful?	❒	❒	❒
16	Are you bothered by dry eyes?	❒	❒	❒
17	Do you feel that you need more time to see things?	❒	❒	❒
18	Do you have vision problems when you participate in traffic (walking, cycling or driving)?	❒	❒	❒
19	Do you have trouble looking for objects and finding objects due to your eyesight?	❒	❒	❒
20	Do you have trouble reading due to your eyesight?	❒	❒	❒

		**Please indicate your answer on a scale of 0 to 10** *(please circle the relevant answer)*
21	To what extent do you experience limitations in daily life due to the above mentioned problems with eyesight?<br>*0 = no limitations*<br>*10 = very severe limitations*	0 1 2 3 4 5 6 7 8 9 10

	**Yes**	**No**
Would you appreciate advice, assessment and/or rehabilitation for the abovementioned complaints?	❒	❒

Please check whether you answered all questions.
One answer must be ticked for each question.

Thank you very much. This is the end of the questionnaire.

Huizinga F, Heutink J, de Haan GA, van der Lijn I, van der Feen FE, Vrijling ACL, Melis-Dankers BJM, de Vries SM, Tucha O, Koerts J. The development of the Screening of Visual Complaints questionnaire for patients with neurodegenerative disorders: evaluation of psychometric features in a community sample. PloS One. 2020
