# Supplementary material for: The development of the Screening of Visual Complaints questionnaire for patients with neurodegenerative disorders: Evaluation of psychometric features in a community sample
Source: PLoS One. 2020 Apr 29;15(4):e0232232. doi: 10.1371/journal.pone.0232232 (PMC7190154; doi:10.1371/journal.pone.0232232)
Supplement: S3 Appendix — (PDF) [file pone.0232232.s003.pdf]

S3 Appendix. Results of convergent validity, divergent validity, test-retest reliability and the relation with participation in daily life for the data without SVC zero scores.

Table 1. Convergent validity of the SVC with the CVC-q and the NEI-VFQ-25.

	SVC total score	1. Diminished visual perception	2. Altered visual perception	3. Ocular discomfort
CVC-q total score	0.81*	0.80*	0.47	0.21
NEI-VFQ-25[a] – total score	-0.68*	-0.64*	-0.37	-0.32
General health[b]	-0.32	-0.30	-0.22	-0.14
General vision[b]	-0.53*	-0.52*	-0.34	-0.16
Near activities	-0.53*	-0.55*	-0.27	-0.13
Distance activities	-0.54*	-0.52*	-0.32	-0.22
Driving	-0.41	-0.42	-0.18	-0.14
Peripheral vision[c]	-0.41	-0.38	-0.31	-0.17
Color vision[c]	-0.21	-0.20	-0.19	-0.06[d]
Ocular pain[b]	-0.43	-0.32	-0.23	-0.56*
Role difficulties	-0.49	-0.48	-0.27	-0.15
Dependency	-0.30	-0.28	-0.25	-0.15
Social functioning	-0.34	-0.32	-0.31	-0.10
Mental health	-0.53*	-0.52*	-0.33	-0.18

All p-values <0.001 (except for [d], p=0.053)

SVC, Screening of Visual Complaints questionnaire; CVC-q, Cerebral Visual Complaints questionnaire; NEI-VFQ-25, National Eye Institute Visual Function Questionnaire-25

[a] In contrast to the SVC and the CVC-q, a higher score on the NEI-VFQ-25 questionnaire indicates better visual function; [b] Subscale composed of two items; [c] Subscale composed of a single item

* Correlations of high strength according to Cohen's criteria

Table 2. Divergent validity of the SVC with the BRIEF-A, the FEDA, the DASS-21 and the SIMS.

	SVC total score	1. Diminished visual perception	2. Altered visual perception	3. Ocular discomfort
BRIEF-A – total score	0.26[#]	0.23[#]	0.22[#]	0.09[#,a]
Metacognition	0.24[#]	0.21[#]	0.19[#]	0.09[#, b]
Behavioral regulation	0.25[#]	0.21[#]	0.21[#]	0.08[#, c]
FEDA	0.38	0.35	0.29[#]	0.15[#]
DASS-21 – total score	0.31	0.26[#]	0.26[#]	0.16[#]
Depression	0.23[#]	0.20[#]	0.21[#]	0.09[#]
Anxiety	0.33	0.26[#]	0.27[#]	0.24[#]
Stress	0.23[#]	0.20[#]	0.20[#]	0.14[#]

SIMS	0.27[#]	0.24[#]	0.22[#]	0.19[#]

All p-values <0.001 (except for [a], p=0.002; [b], p=0.004; [c], p=0.005)

SVC, Screening of Visual Complaints questionnaire; BRIEF-A, Behavior Rating Inventory of Executive Function-Adults; FEDA, Questionnaire for Experiences of Attention Deficits (*Fragebogen erlebter Defizite der Aufmerkzamkeit*); DASS-21, Depression Anxiety Stress Scale–21; SIMS, Structured Inventory for Malingered Symptomatology

[#] Correlations of low strength according to Cohen's criteria

Table 3. Internal consistency of the SVC

Factor	Number of items	Cronbach's alpha [CI]
1. Diminished visual perception	11	0.80 [0.79-0.82]
2. Altered visual perception	6	0.61 [0.57-0.64]
3. Ocular discomfort	2	0.46 [0.40-0.52]
**SVC Total**	**19**	**0.82 [0.81-0.84]**

CI= Confidence Interval

Table 4. Test-retest reliability of the SVC

Factor	Number of items	ICC [CI]
1. Diminished visual perception	11	0.80 [0.69-0.88]
2. Altered visual perception	6	0.75 [0.60-0.84]
3. Ocular discomfort	2	0.62 [0.43-0.76]
**SVC Total**	**19**	**0.77 [0.63-0.86]**

ICC = Intraclass correlation coefficient; CI= Confidence Interval

The weighted Kappa coefficient for item 21 of limitations in daily life was 0.65.

Table 5. Relation of the SVC with self-related limitation in daily life and the USER

	**SVC total score**
SVC limitations in daily life	0.63**
USER subscale frequency	-0.04
USER subscale satisfaction	-0.19**

SVC, Screening of Visual Complaints questionnaire; USER, Utrecht Scale for Evaluation of Rehabilitation-Participation

** p<0.001
